# Heme oxygenase-1 induction attenuates imiquimod-induced psoriasiform inflammation by negative regulation of Stat3 signaling

**DOI:** 10.1038/srep21132

**Published:** 2016-02-19

**Authors:** Bin Zhang, Sijing Xie, Zhonglan Su, Shiyu Song, Hui Xu, Gang Chen, Wangsen Cao, Shasha Yin, Qian Gao, Hongwei Wang

**Affiliations:** 1 Center for Translational Medicine and Jiangsu Key Laboratory of Molecular Medicine, Medical School of Nanjing University, Nanjing 210093, P.R. China; 2 Department of Dermatology, the First Affiliated Hospital of Nanjing Medical University, Nanjing 210029, P.R. China; 3 Department of Esthetic Plastic Surgery, The First Affiliated Hospital of Nanjing University of TCM, Nanjing, Jiangsu 210029, P.R. China; 4 Central Laboratory, Nanjing Chest Hospital, Medical School of Southeast University, Nanjing, Jiangsu, 210029, China; 5 Nanjing Stomatology Hospital, Medical School of Nanjing University, Nanjing, Jiangsu, 210093, China

## Abstract

Heme oxygenase-1 (HO-1), a stress-inducible protein with a potential anti-inflammatory effect, plays an important role in skin injury and wound healing. However, the function of HO-1 in cutaneous inflammatory diseases, such as psoriasis, remains unknown. The abnormal activation of Stat3, a known transcription factor that induces inflammation and regulates cell differentiation, is directly involved in the pathogenesis and development of psoriasis. Hence, targeting Stat3 is potentially beneficial in the treatment of psoriasis. In this study, HO-1 activation significantly alleviated the disease-related pathogenesis abnormality. To determine the mechanism by which HO-1 exerts immune protection on Th17-related cytokines, IL6/IL22-induced Stat3 activation was significantly suppressed, accompanied by decreased cell proliferation and reversed abnormal cell proliferation. Importantly, HO-1-induced Stat3 suppression was mediated through the activation of protein tyrosine phosphatase SHP-1. Overall, our study provides direct evidence indicating that HO-1 might be a useful therapeutic target for psoriasis. SHP-1-mediated suppression of Stat3 activation after HO-1 activation is a unique molecular mechanism for the regulation of Stat3 activation.

Psoriasis, which affects approximately 3% of the world’s population, is one of the most common inflammatory skin disorders^1^. This disease is defined by a series of linked cellular changes in the skin: epidermal keratinocyte hyperplasia, vascular hyperplasia and ectasia, and inflammatory cell infiltration, especially in T cells in the affected skin. The pathogenesis of psoriasis remains incompletely understood, and further understanding of the molecular mechanisms underlying the development of psoriasis may help in identifying novel targets for pharmacological intervention of this disease^2^.

Previous studies demonstrated that the activation of signal transducer and activator of transcription 3 (Stat3) signaling in keratinocytes directly contributes to the pathogenic development of psoriatic lesions^3^. Stat3 is a latent cytoplasmic protein that conveys signals to the nucleus upon stimulation by interleukin (IL)-6, IL-11, epidermal growth factor (EGF), and many other cytokines or growth factors, leading to the transcriptional activation of downstream genes. Stat3 performs critical functions in cell activities, including cell proliferation, migration, survival, and oncogenesis^4^,^5^. Clinically, Stat3 is a novel target involved in the development of psoriasis; Stat3 is constitutively active in psoriasis, and its expression is correlated with the severity of the disease^6^. Inhibition of Stat3 activation can effectively reduce the pathological symptoms of psoriasis^7^. Therefore, Stat3 has been recognized as a therapeutic target for psoriasis treatment. Stat3 is activated by the phosphorylation of tyrosine 705 by receptor and non-receptor protein tyrosine kinases, such as EGF receptor kinase, Src, Janus-activated kinase (JAK), and extracellular signal-regulated kinase^8^. The negative regulatory molecules of Stat3 include the suppressor of cytokine signaling (SOCS), Src homology phosphatase-1 (SHP-1), protein inhibitor of activated Stat (PIAS), and protein tyrosine phosphatases (PTPases)^9^.

Heme oxygenase (HO) is the rate-limiting enzyme in heme degradation. In addition to its obvious function in iron metabolism, previous studies indicated that HO is important for cellular protection against inflammation and oxidative stress^10^. HO has three isoforms. The inducible isoform HO-1 is involved in wound repair and resolution of inflammatory response. HO-1 expression is associated with epidermal differentiation in normal skin. Moreover, increased HO-1 expression has been demonstrated in psoriatic lesions^11^. However, the pathogenesis of HO-1 in psoriasis still needs to be defined.

This paper presents data supporting a novel function for HO-1 in psoriasis. The therapeutic effects of HO-1 on psoriatic lesional skin were demonstrated using an imiquimod (IMQ)-induced psoriatic mouse model, and a crosstalk between the Stat3 and HO-1 pathways was observed in cultured keratinocytes. HO-1 overexpression significantly inhibited Stat3 activation, and this inhibition was dependent on SHP-1, a PTPase. The negative regulation of Stat3 decreased keratinocyte proliferation and reversed its abnormal differentiation. To date, the correlation between HO-1 induction/Stat3 activation and the therapeutic effect on experimental psoriasis has not been reported. Our data provide direct experimental evidence of the possibility of targeting HO-1/Stat3 for psoriasis therapy.

## Results

### Epidermal keratinocytes of human psoriatic lesions show activated Stat3 and HO-1

**Figure 1:**
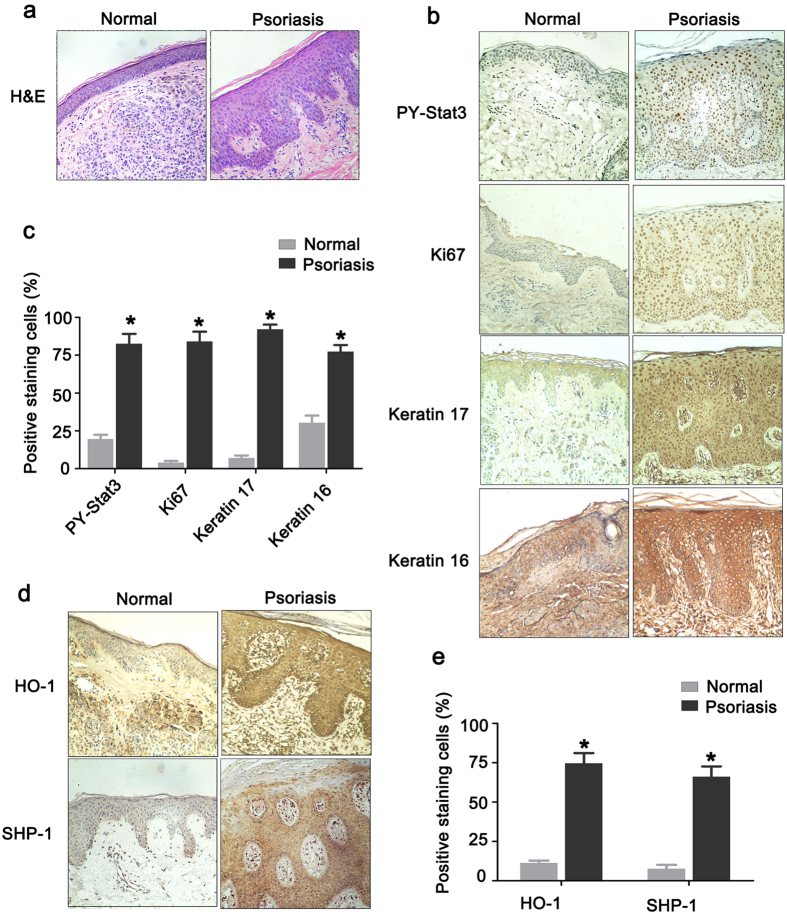
High levels of Stat3 activation and increased HO-1 expression are preferentially associated with abnormal keratinocyte differentiation and overproliferation in psoriatic lesional skin. Formalin-fixed paraffin-embedded tissue sections from psoriasis patients and healthy individuals were examined by histopathological assay. (**a**) H&E staining of normal skin and psoriatic lesional skin. (**b**) Representative IHC staining for PY-Stat3 (Tyr705), Ki67, keratin 16, or keratin 17 Abs in serial sections of psoriatic lesional skin or healthy controls. (**c**) The statistical analysis of IHC staining for PY-Stat3 (Tyr705), Ki67, keratin 16, or keratin 17 in psoriatic lesional skin or healthy controls. (**d**) Representative IHC staining for HO-1 or SHP-1 in serial sections of psoriatic lesional skin or healthy controls. (**e**) The statistical analysis of IHC staining for HO-1 or SHP-1 in serial sections of psoriatic lesional skin or healthy controls. Magnification: ×100. *represents P < 0.05, which indicates a statistically significant difference.

Skin inflammation is among the major features of psoriasis. The pathological features of psoriasis were initially characterized by immunohistochemical staining^12^. In accordance with previous studies, H&E staining showed that psoriatic lesional skin exhibited severe and significant epidermal thickening and parakeratosis, accompanied by increased lymphocyte infiltration (Fig. 1a). The expression patterns of several crucial molecules, including Ki67, keratin 16, and keratin 17, were analyzed to further investigate the molecular pathological features. Figure 1b shows significantly increased expression of Ki67, a key protein marker associated with cell proliferation. Tissue distribution analysis showed that Ki67-positive stained cells in normal skin were located mainly in the basal/germinal layer of the skin, whereas Ki67-positive stained cells in psoriatic lesional skin were widely distributed, covering the entire skin epidermal layer. These results indicate that the keratinocytes from psoriatic patients underwent enhanced cell proliferation. The expression levels of keratin family proteins, such as keratin 16 and keratin 17, which are markers for hyperproliferation and abnormal differentiation in keratinocytes^13^, also significantly increased.

Stat3, an important signaling pathway in biological activities, including cell proliferation, migration, survival, and oncogenesis, has been assumed involved in the development of psoriasis^3^. The results of Stat3 activation measurement showed that numerous keratinocytes were positively stained by Stat3 phosphorylation in Tyr705 (PY-Stat3) in human psoriatic lesions (Fig. 1b). PY-Stat3 staining was mainly located in keratinocyte nuclei, indicating the constitutive activation of Stat3 in these cells. Statistical analysis indicated a good correlation between PY-Stat3 expression and the density of Ki-67^+^, keratin 16^+^, or keratin 17^+^ keratinocytes in the epidermis (r = 0.95, r = 0.861, and r = 0.897, respectively). These findings indicate that the expression of Ki67, keratin 16, and keratin 17 were features of psoriasis, and Stat3 activation may differentially influence and control their expression. Further measurements of the expression of the Stat3-specific inhibitory molecule SHP-1 showed that SHP-1 expression in psoriatic lesional skin significantly increased. Thus, PTPase-related negative regulatory molecules were activated in psoriatic lesional skin to restrict excessive Stat3 activation (Fig. 1c).

### HO-1 activation inhibits Th17 cytokine-induced keratinocyte proliferation

**Figure 2:**
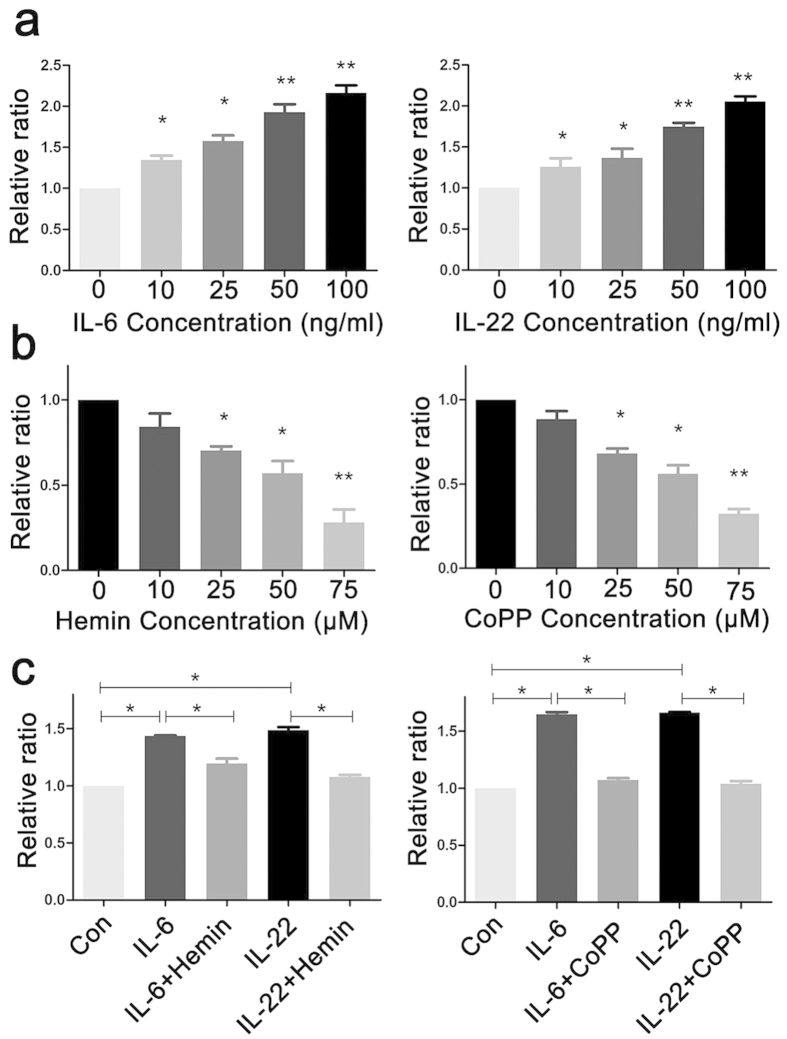
Pretreatment of HaCaT cells with HO-1 activators Hemin or CoPP attenuates Th17 cytokine-induced keratinocyte hyperproliferation. (**a**) HaCaT cells were seeded in 96-cell plates, and stimulated with 10, 25, 50, and 100 ng/mL IL-6 or IL-22 for 24 h before measuring cytokine-induced keratinocyte proliferation using MTS assay, which was based on the cell viability assay. (**b**) HaCaT cells were seeded in 96-cell plates, and stimulated with 10, 25, 50, and 75 nM Hemin or CoPP for 24 h before measuring cytokine-induced keratinocyte proliferation using MTS assay, which was based on the cell viability assay. (**c**) HaCaT cells were stimulated with 25 ng/mL IL-6/IL-22 for 24 h in the presence or absence of 25 μM Hemin or CoPP. Total cell numbers were assessed based on the cell viability assay detected using the MTS methods. *represents P < 0.05, which indicates a statistically significant difference. **represents P < 0.005.

HO-1, the rate-limiting enzyme in heme metabolism, performs multiple functions in cytoprotection and immune regulation^14^. HO-1 expression in the skin was measured to elucidate the effects of HO-1 on psoriasis. Immunochemistry staining showed that psoriatic lesional skin had significantly more HO-1-positive stained cells, and most HO-1-positive stained cells were keratinocytes widely distributed throughout the skin epithelium (Fig. 1c). The influence of HO-1 on keratinocyte growth and differentiation was then examined to address the function of HO-1 in psoriasis. A previous report suggested that the abnormal activation of T cells, especially the Th17 cell subset, contributes to the pathogenesis of psoriasis, and Th17 cell-related cytokines, including IL-17, IL22, and IL-6, are responsible for the altered proliferation and differentiation of keratinocytes and the induction of Stat3 signaling^15^. To test these effects, HaCaT cells were exposed to various IL-6 and IL-22 doses for 24 h, and cytokine-induced cell proliferation was measured using MTS cell proliferation assay. The results showed that the exposure of HaCaT cells to 10–100 ng/mL IL-6 or IL-22 for 24 h significantly promoted cell proliferation in a dose-dependent manner (Fig. 2a). By contrast, HO-1 activator Hemin or CoPP significantly inhibited HaCaT cell proliferation at the dose of 25 μM (Fig. 2b). HaCaT cells that were simultaneously treated for 24 h with IL-6/IL-22 (25 ng/mL) and HO-1 activators, such as Hemin or CoPP (25 μmol/L), significantly decreased cell proliferation (Fig. 2c). These results suggest that HO-1 activation can block Th17 cytokine-induced cell proliferation in HaCaT cells.

### HO-1 activation can inhibit Stat3 phosphorylation and reverse Stat3-controlled aberrant keratinocyte differentiation

**Figure 3:**
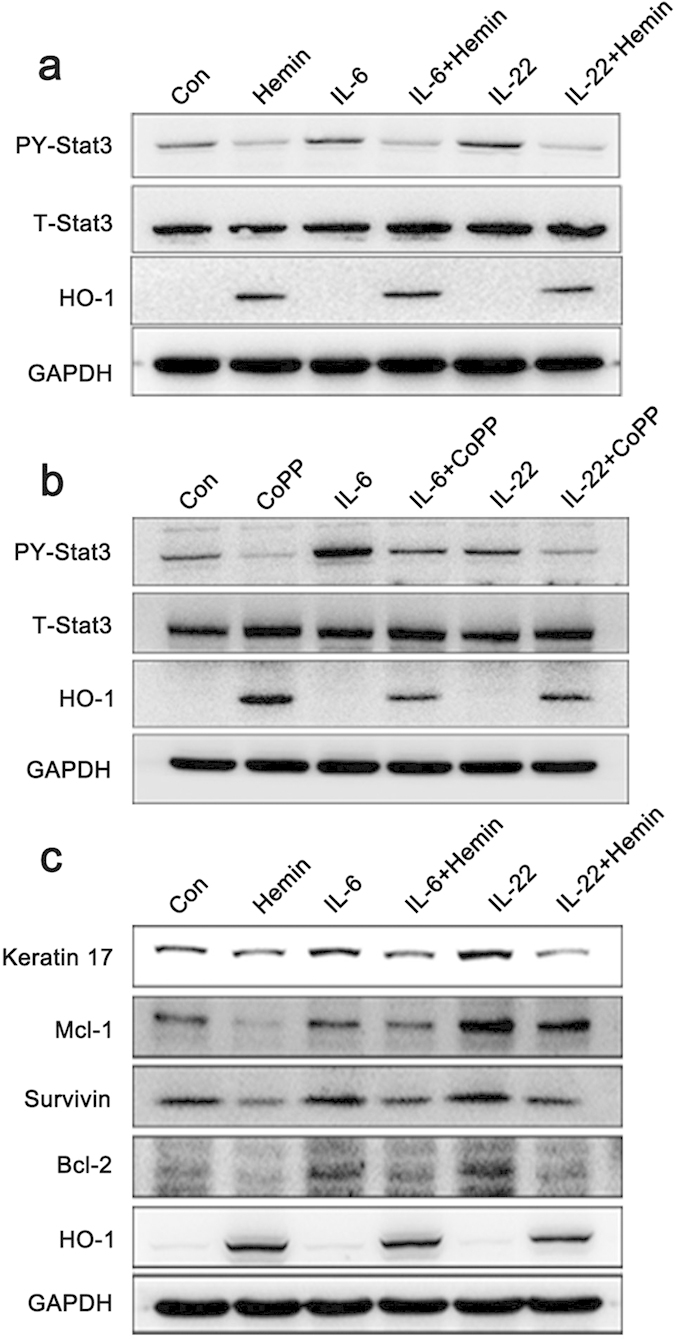
HO-1 activation in HaCaT cells inhibits Th17 cytokine-induced Stat3 phosphorylation and downstream gene expression. (**a**) HaCaT cells were pretreated with 25 μM Hemin for 24 h before exposure to 25 ng/mL IL-6 or IL-22 for 30 min. Whole-cell extracts were collected, and Stat3 activation (PY-Stat3) was determined. (**b**) HaCaT cells were pretreated with 25 μM CoPP for 24 h before exposure to 25 ng/mL IL-6 or IL-22 for 30 min. (**c**) HaCaT cells were treated with 25 μM Hemin for 12 h before exposure to 25 ng/mL IL-6 or IL-22 for 24 h. Whole-cell extracts were collected, and the expression levels of Stat3 downstream genes (Mcl-1, survivin, and Bcl-2) and abnormal keratin (keratin 16 and keratin 17) were determined by Western blot. The blots shown are derived from multiple gels. The gels were run under the same experimental conditions. The membrane was cut based on molecular weight and probed with antibody of interest. Band of interest is indicated with an arrow. All full-length blots are presented in Supplementary Fig. 1.

The inhibitory mechanism of HO-1 in keratinocyte proliferation was further examined. Stat3 underwent activation through Tyr705 phosphorylation, followed by dimerization and nuclear translocation; stimulation of HaCaT with IL-6 or IL-22 caused rapid Stat3 Tyr705 (PY-Stat3) phosphorylation^16^. However, HaCaT cells pretreated with Hemin almost completely blocked this effect (Fig. 3a). Another HO-1 activator, CoPP, was used in this study to confirm the findings. Similar results were obtained, suggesting that Th17 cytokine-induced Stat3 activation in keratinocytes can be abrogated via HO-1 activation (Fig. 3b).

The activation of Stat3 regulates the expression of several downstream genes, including several cell-cycle and anti-apoptosis proteins, such as survivin, cyclin D1, and the Bcl family^17^. In our study, the exposure of HaCaT cells to Th17-related cytokines, such as IL-6 and IL-22, significantly increased Stat3 downstream gene expression, whereas the pretreatment of HaCaT cells with HO-1 activator Hemin considerably decreased the expression of Mcl-1, survivin, and Bcl-2 (Fig. 3c). Stat3 activation in keratinocytes is linked to abnormal cell differentiation, which is characterized by the increased expression of keratin 17 and keratin 16; these two are markers of hyperproliferation expressed only in psoriatic lesional epidermis^18^. Our results showed that IL-6- and IL-22-induced Stat3 activation significantly increased keratin 17 expression, but keratinocyte pretreatment with Hemin substantially decreased keratin 17 expression (Fig. 3c). HO-1 activation appeared to influence keratinocyte differentiation and proliferation, and Stat3 signaling mediated these effects.

### Regulation of HO-1 expression via RNA interference (RNAi) or recombinant plasmids alters Stat3 activation and downstream gene expression

**Figure 4:**
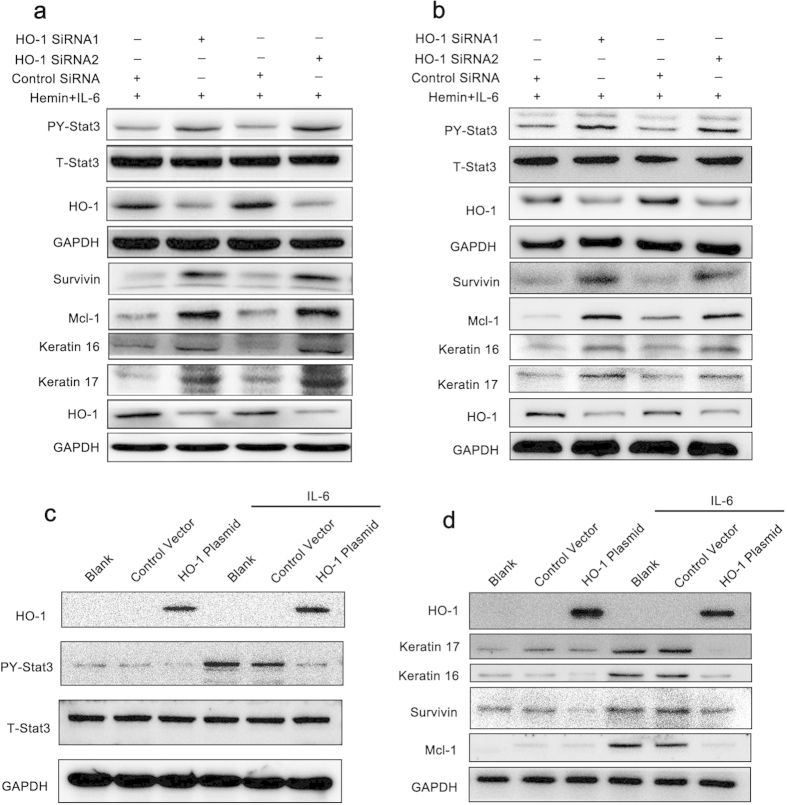
Altered HO-1 expression levels by recombinant expression or RNAi also influence Stat3 activation in keratinocytes. (**a**) Altered HO-1 expression by RNAi increased Stat3 phosphorylation and downstream genes expression in HaCaT cells. (**b**) Altered HO-1 expression by RNAi increased Stat3 phosphorylation and downstream genes expression in A431 cells. In the RNAi experiments, HaCaT or A431 cells were cultured to 80% confluency in six-well plates. These cells were transfected with 100 nM specific HO-1 siRNA or control siRNA and treated with 25 μM Hemin for 24 h before exposure to 25 ng/mL IL-6 for 30 min. Stat3 phosphorylation levels and downstream proteins were determined by Western blot. (**c**) Enhanced HO-1 expression using the transfected recombinant plasmids decreased Stat3 activation in the presence or absence of IL-6 stimulus. (**d**) Enhanced HO-1 expression decreased the expression of Stat3 downstream proteins in the presence or absence of IL-6 stimulus. In gene overexpression experiments, HaCaT cells were transfected with GV230-HO-1 recombinant plasmid for 24 h before exposure to 25 ng/mL IL-6 for 30 min or 24 h, and the Stat3 phosphorylation levels and the expression of Stat3 downstream genes were determined by Western blot. Blots shown are derived from multiple gels. The gels were run under the same experimental conditions. The membrane was cut based on molecular weight and probed with antibody of interest. Band of interest is indicated with an arrow. All full-length blots are presented in Supplementary Fig. 2.

To further analyze the regulatory effect of HO-1 on Stat3, RNAi or gene overexpression was used to alter the HO-1 expression level. Various siRNA sequences were synthesized to fulfill RNAi, as mentioned in the Material and Methods section, and the gene knockdown efficiencies were compared. Figure 4a,b shows that two siRNAs were used in our experiments. When HO-1 was knocked down in HaCaT cells by using siRNAs, the IL-6-induced Stat3 phosphorylation significantly increased compared with the control group. The expression levels of Stat3 downstream genes, such as Survivin and Mcl-1, also considerably increased after 48 h of siRNA treatment (Fig. 4b). Analysis of the gene expression levels of keratin 16 and keratin 17 showed that the knockdown of HO-1 gene expression resulted in the increased expression of keratin 16 and keratin 17 in response to IL-6 treatment (Fig. 4a). The same results were procured by using a human epidermal carcinoma cell line, A431 (Fig. 4a). This result indicates that the knockdown of HO-1 gene expression enhanced Stat3 activation. The HO-1 recombinant plasmid was transferred to HaCaT cells in another experiment, and HO-1 overexpression was achieved (Fig. 4c,d). HO-1 overexpression markedly decreased Stat3 phosphorylation at the basal level, and similar results were observed when the cells were treated with IL-6. These results further confirmed that the increased HO-1 expression in keratinocytes inhibited Stat3 activation.

### HO-1 restrains Stat3 activation through SHP-1 expression upregulation

To further explain the mechanism of HO-1 in the regulation of Stat3 activation, the expression of Stat3 regulatory molecules was examined. PTPases are involved in the negative regulation of JAK/STAT signaling through direct p-JAK2 and p-Stat3 (Tyr 705) dephosphorylation^9^,^19^. Among the PTPases, SHP-1 positively regulates EGF- or IFN-γ-induced Stat1 activation in HeLa cells^20^, but it is usually reported as a negative Stat3 regulator^21^,^22^. Psoriatic lesional skin displayed higher SHP-1 levels than normal skin; hence, the involvement of SHP-1 in Stat3 regulation was assessed.

**Figure 5:**
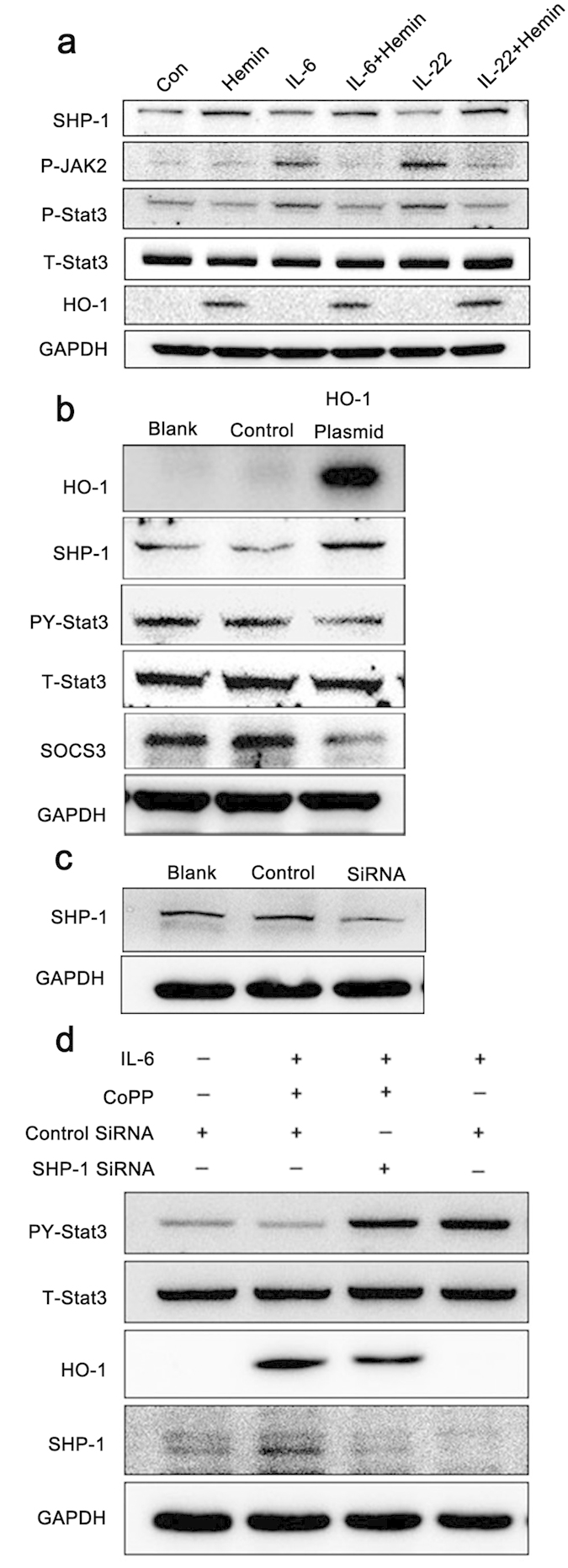
HO-1-induced Stat3 inactivation is mediated by SHP-1. (**a**) HaCaT cells were pretreated with 25 μM Hemin for 24 h before exposure to 25 ng/mL IL-6 or IL-22 for 30 min. SHP-1 expression was assessed and Stat3 activation was detected based on the phosphorylation levels of Stat3 and JAK. (**b**) HaCaT cells were transfected with GV230-HO-1 recombinant plasmid, and the correlation between HO-1 overexpression and SHP-1 activation was assessed by immunoblot assay. (**c**) Western blot analysis of SHP-1 expression after RNAi. (**d**) Knockdown of SHP-1 expression by RNAi completely nullified the HO-1-mediated Stat3 regulatory effects in HaCaT cells. HaCaT cells were transfected with SHP-1 siRNA for 36 h before exposure to 25 μM CoPP for 24 h and 25 ng/mL IL-6 for 30 min. Stat3 activation (PY-Stat3) was determined by Western blot. Blots shown are derived from multiple gels. The gels were run under the same experimental conditions. The membrane was cut based on molecular weight and probed with antibody of interest. Band of interest is indicated with an arrow. All full-length blots are presented in Supplementary Fig. 3.

SHP-1 expression in HaCaT cells was also examined in response to Hemin treatment (Fig. 5a). Hemin treatment for 24 h resulted in significantly elevated SHP-1 expression. The increased SHP-1 expression was correlated with attenuated JAK2 and Stat3 phosphorylation in the presence or absence of IL-6/IL22 stimulus, suggesting that SHP-1 was definitely involved in HO-1-regulated Stat3 activation. To exclude the possible nonspecific effects of Hemin on HO-1 regulation, transgenic approach was used for HO-1 overexpression in HaCaT cells by constructing HO-1 recombinant plasmids. Notably, transfection of HaCaT cells with HO-1 recombinant expression plasmids for 24 or 48 h significantly upregulated HO-1 expression. Under HO-1 overexpressed conditions, IL-6-induced Stat3 activation (PY-Stat3, Tyr705) in HaCaT cells significantly decreased, which was accompanied by considerably increased SHP-1 expression (Fig. 5b). By contrast, knockdown of SHP-1 expression in HaCaT cells almost completely nullified the regulatory function of HO-1 in Stat3 activation (Fig. 5c). These results indicate that HO-1-mediated Stat3 inhibition may rely on upregulated SHP-1 expression.

**Figure 6:**
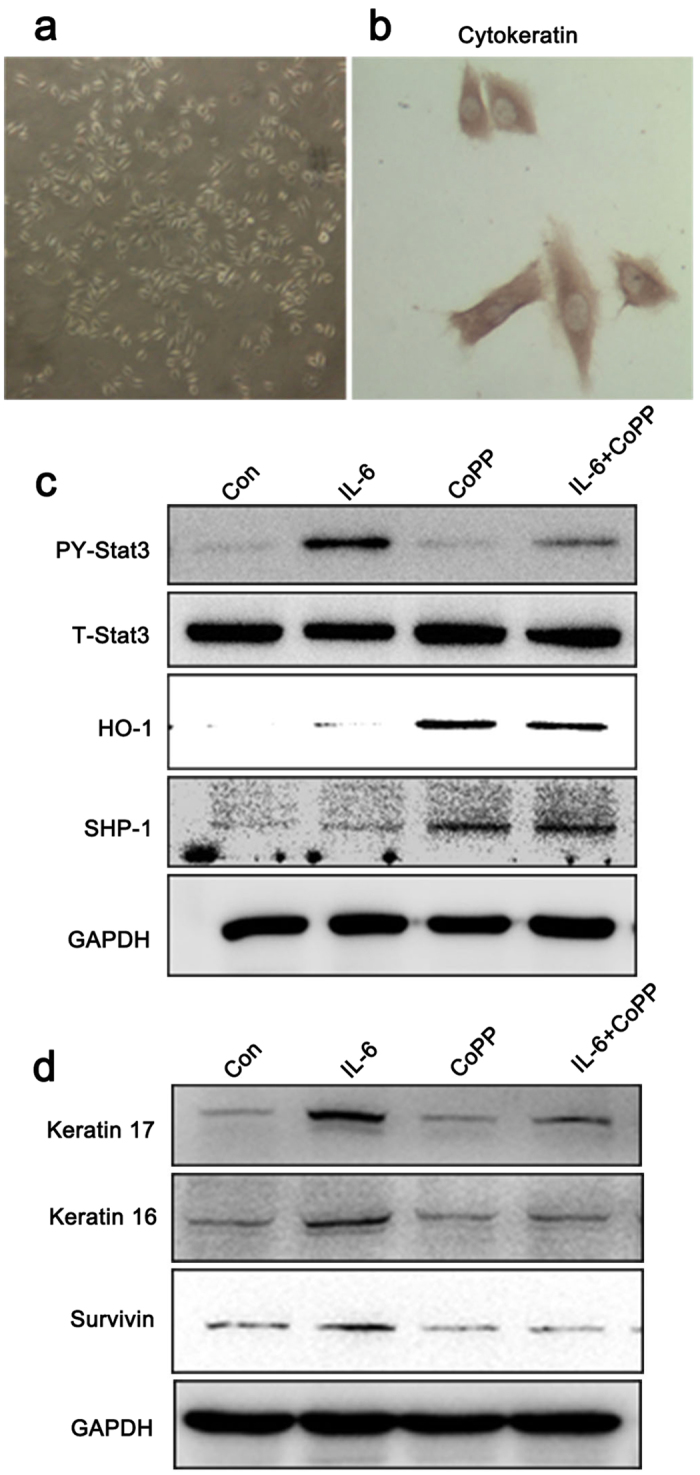
HO-1 inhibited Stat3 activation in normal human keratinocyte through SHP-1 expression. (**a**) The morphology of cultured primary human keratinocytes was observed under phase–contrast microscope. Magnification: ×100. (**b**) Immunocytochemistry staining of isolated human keratinocytes with cytokeratin (AE1/AE3) Ab. Magnification: ×400. (**c**) NHKC were pretreated with 25 μM CoPP for 24 h before exposure to 25 ng/mL IL-22 for 30 min. Whole-cell extracts were collected, and Stat3 activation (PY-Stat3), HO-1 and SHP-1 expression were determined. (**d**) NHKC were pretreated treated with 25 μ Hemin for 12 h before exposure to 25 ng/mL IL-22 for 24 h. Whole-cell extracts were collected, and the expression levels of Stat3 downstream genes survivin and abnormal keratin (keratin 16 and keratin 17) were determined by Western blot. Blots shown are derived from multiple gels. The gels were run under the same experimental conditions. The membrane was cut based on molecular weight. All full-length blots are presented in Supplementary Fig. 4.

To further demonstrate the inhibition effect of HO-1 on Stat3 activation through SHP-1, we used normal human keratinocytes (NHKCs). NHKCs were isolated from the skin of healthy individuals through plastic surgery, as mentioned in the Material and Methods section. Immunochemistry results showed positive staining of the main marker of keratinocytes, that is, cytokeratin (AE1/AE3), in isolated cells (Fig. 6a). IL-6 treatment stimulated the phosphorylation of Stat3 Try 705 and the expression of keratin 16, keratin 17, and survivin. By contrast, pretreatment with CoPP stimulated the expression of HO-1 and SHP-1 and inhibited Stat3 activation and abnormal keratin expression (Fig. 6b,c).

### Therapeutic effect of HO-1 activator in IMQ-induced psoriatic mouse model

**Figure 7:**
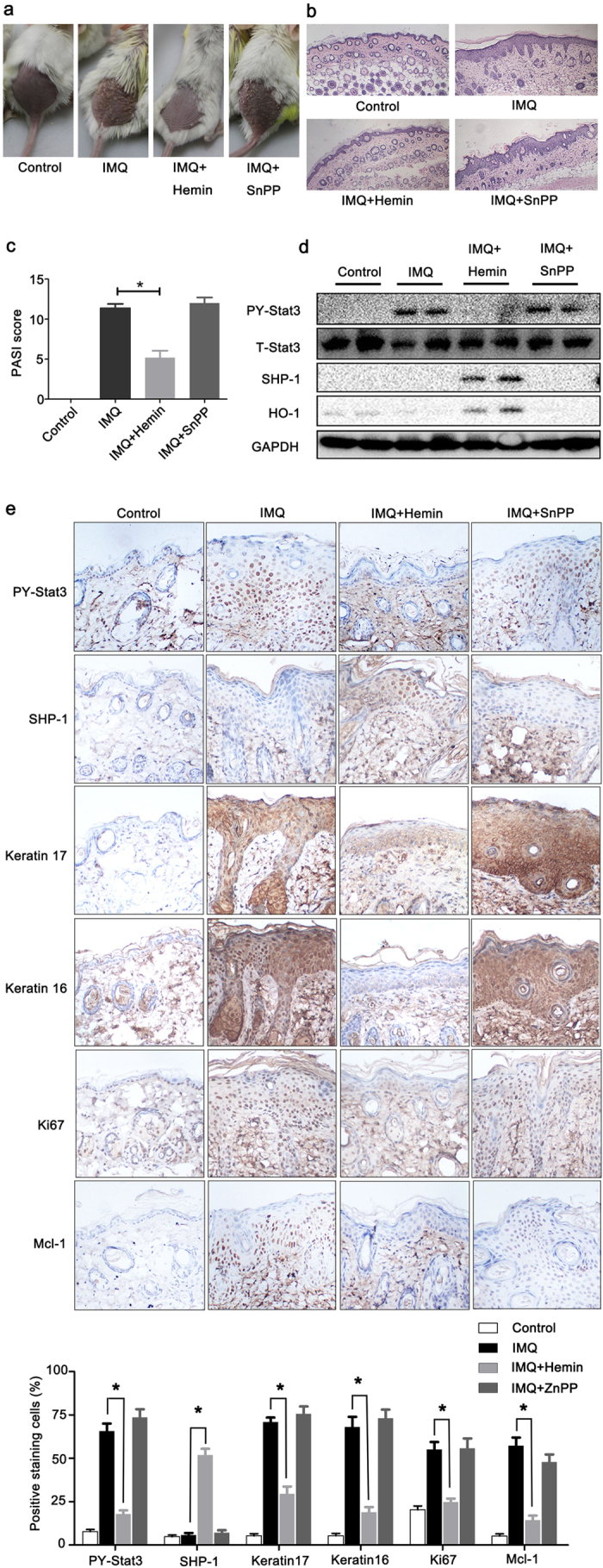
Therapeutic effect of HO-1 activators on the IMQ-induced psoriasis mouse model. Topical treatment of IMQ cream (5%) for 7 d on the shaved back skins of BALB/C mice. Skin samples were collected before euthanasia. (**a**) Pictures of the back skin of the mice before euthanasia. (**b**) Pathological analysis with H&E staining of the back skin samples of mice from different treatment groups. (**c**) PASI score indicated disease severity (n = 5). (**d**) Back skin samples were lysed, and the whole protein was collected. Western blot was performed to determine the levels of PY-Stat3, HO-1, and SHP-1. Blots shown are derived from multiple gels. The gels were run under the same experimental conditions. The membrane was cut based on molecular weight. All full-length blots are presented in Supplementary Fig. 5. (**e**) Immunochemistry staining of the harvested skin lesion samples with Abs of PY-Stat3, Stat3 downstream protein Mcl-1, proliferation marker Ki67, and abnormal keratin (keratin 16 and keratin 17). *represents P < 0.05, which indicates a statistically significant difference.

HO-1 was hypothesized as a feasible therapeutic target of psoriasis because HO-1 counteracted Stat3-linked aberrant differentiation and proliferation in keratinocytes. Topical application of IMQ, a TLR7/8 ligand and potent immune activator, induces and exacerbates psoriasis^23^. IMQ-induced dermatitis in mice is proposed as one of the most promising and recently established animal models to analyze the pathogenic mechanism in psoriasis-like dermatitis^24^. This model was implemented as previously described, and several different chemical modulators of HO-1 were used in the present study. The application of IMQ on the shaved back skin of BALB/c mice produced a psoriatic-like lesion, exhibiting signs of erythema, scaling, and thickening. Topical Hemin treatment significantly attenuated the severity of skin lesions in animals. Moreover, epidermal hyperplasia significantly decreased, and the amount of dermal-infiltrated inflammatory cells was reduced. The group that received HO-1 inhibitor SnPP treatment showed no significant changes in the scoring parameters, such as skin thickness or skin scaling (Fig. 7a). Histological examination with H&E staining showed increased epidermal thickening in the IMQ group and SnPP treatment group, compared with that in the control animals or Hemin treatment group (Fig. 7b). The score of psoriasis area and the severity index showed a consistent change (Fig. 7c). Western blot analysis was performed using mouse back skin biopsy samples to further assess the effect of the chemical modulators of HO-1 on psoriasis. The results showed that Hemin administration significantly elevated the expression levels of HO-1 and SHP-1 and considerably reduced Stat3 phosphorylation of Tyr705 (Fig. 7d) in the skin epidermis of psoriatic mice. The expression and tissue distributions of Stat3 downstream molecules were measured by immunochemistry staining. Mcl-1, keratin 16, keratin 17, and proliferation marker Ki-67 were significantly enhanced after IMQ treatment, but Hemin administration substantially nullified these effects. Administration of SnPP, a HO-1 specific inhibitor, cannot alleviate the disease severity based on the pathological features of skin lesions (Fig. 7e). These results suggest that HO-1 activation may be an important approach for psoriasis treatment.

## Discussion

Psoriasis is a chronic inflammatory skin disease characterized by epidermal hyperplasia (acanthosis), leukocyte infiltration into both the dermis and epidermis, and dilation and growth of blood vessels^25^. At present, the pathogenetic mechanism of psoriasis remains largely unknown. Although psoriasis has a genetic component, various environmental factors have been suggested to aggravate psoriasis, including oxidative stress. Skin is a major target of oxidative stress mainly because of reactive oxygen species from the environment and skin metabolism; additionally, chronic inflammation and skin tissue damage are partly caused by prolonged exposure to excessive amounts of reactive oxygen metabolites, causing oxidative stress^26^. HO is a 32 kDa stress protein that mediates the degradation of heme to ferrous iron, carbon monoxide, and biliverdin/bilirubin, which are potential antioxidants. The HO family consists of three different isoforms (HO-1, HO-2, and HO-3), HO-1 is the inducible rate-limiting enzyme in heme catabolism^27^. HO-1 is often viewed as a cytoprotective gene because of its multiple cytoprotective effects. HO-1 is mainly expressed in skin keratinocytes and is involved in wound repair and inflammatory response^28^. However, the function of HO-1 in psoriasis remains unclear. The IMQ-induced psoriatic mouse model and cultured keratinocytes were used to demonstrate the therapeutic effects of HO-1 on psoriasis, and the results suggest that the anti-psoriasis function of HO-1 was largely attributed to its effect on Stat3.

The Stat3 signaling pathway is activated by multiple cytokines and growth factors, and Stat3 activation is involved in modulating cell proliferation, differentiation, and apoptosis^29^. In normal physiological conditions, Stat3 activation is tightly regulated and usually transient. However, in psoriasis, Stat3 was consistently activated in epidermal keratinocytes, which was assumed to recapitulate persistent wound healing reaction. Stat3C transgenic mice, in which keratinocytes express constitutively active Stat3 (PY-Stat3), developed psoriasis-like skin lesions, suggesting that Stat3 activation caused disease pathogenesis^30^. The present results also confirmed that consistent Stat3 activation was a crucial pathogenesis event of psoriasis because Stat3 was constitutively activated in psoriatic lesional skin.

Keratinocyte proliferation and differentiation in normal skin are tightly coupled to maintain normal architecture in continually renewing tissues. Only the basal layer of keratinocytes in this study was stained with Ki67, a cell proliferation marker. We observed a significant increase in Ki67 expression in psoriatic lesional skin, indicating that keratinocytes proliferated and the basal cell layer expanded in psoriasis. We also observed the abnormally enhanced expression of keratin molecules, including keratin 16 and keratin 17, which function as markers of hyperproliferation and aberrant differentiation^18^,^31^,^32^. The current study supports the conclusion that keratin 16 and keratin 17 expression in keratinocytes were upregulated by Stat3-dependent mechanisms, accelerating the development of psoriasis.

Psoriasis is a T-cell-mediated disease, and distinct T-cell infiltration can be detected in lesions from psoriasis patients. Stat3 is essential for Th17 differentiation and the inhibition of Treg cell functions; however, increased Th17 cell infiltration and reduced Treg function have also been reported in psoriasis patients^33^. Previous studies showed that Th17 cell-related cytokines, such as IL-6 and IL-22, are highly expressed in psoriasis, and their pleiotropic effects include epidermal keratinocyte hyperplasia and stimulation of blood vessel formation^34^. Therefore, the inhibition of Th17 signaling may be an effective treatment for psoriatic epidermal hyperplasia^35^. The treatment of HaCaT cells with IL-6 and IL-22 resulted in increased cell proliferation and abnormal cell differentiation, which were marked by the enhanced expression of keratin 16 and keratin 17. The induction of HO-1 activation using HO-1 activator Hemin or CoPP significantly inhibited IL-6-/IL22-induced keratinocyte proliferation and abnormal differentiation. These findings indicate that HO-1 activation was a therapeutic target for hyperproliferative skin diseases, such as psoriasis. The results also confirmed that Stat3 phosphorylation, the most well-characterized signaling event following IL-6 and IL-22 treatment in keratinocytes, was inhibited by HO-1 induction.

**Figure 8:**
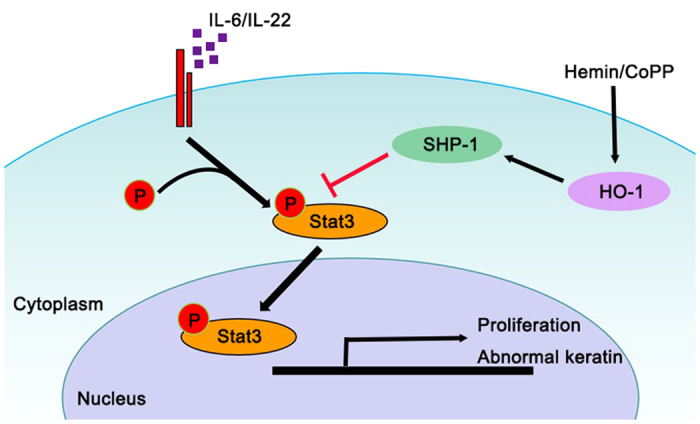
The diagram shows the Proposed Molecular mechanism of HO-1 mediated Stat3 inhibition. Persistent actived Stat3 promotes hyperproliferation and abnormal keratin expression in the keratinocytes of psoriasis patients, treatments with Hemin/CoPP induce HO-1 expression, which enhance the expression of SHP-1 in cell plasma, this result in the inhibition of Stat3 phosphorylation and the decrease of downstream genes expression.

Stat3 acts as a positive regulator for the development of psoriasis; however, the molecular mechanism of Stat3 activation in keratinocytes is unclear. Stat3 signaling is normally tightly regulated by several inhibitory molecules, including SOCS, PIAS, and PTPases^36^. To elucidate the mechanism of the suppressive action of HO-1 on Stat3 activation, we also investigated the expression of SHP-1 PTPases in response to HO-1 induction. The results showed that HO-1 induction was associated with increased SHP-1 expression. The expression of Stat3 downstream molecules, including SOCS3, MC-1, and survivin, was significantly inhibited by Stat3 inactivation. Our data indicate that HO-1 mediated the enhanced expression of SHP-1, which interferes with Stat3 signaling, resulting in an almost complete inhibition of Th17 cytokine-mediated keratinocyte proliferation and abnormal differentiation (Fig. 8).

To test the therapeutic effect of HO-1 activator *in vivo*, IMQ-induced psoriasis mouse models were established through the topical application of 5% IMQ cream. Histopathological evaluation of the mouse skin sections confirmed that mice develop a wide range of abnormalities characterized by epidermal thickening because of hyperkeratosis, infiltration of immune cells in dermis and epidermis, parakeratosis, and neovascularization. These symptoms showed a hallmark of the disease in humans that involved in the IL-23/IL17 cytokine axis^37^ Targeting the HO-1 molecules with the HO-1 activator Hemin effectively blocked the development of epidermal hyperplasia and dermal inflammation in this study. By contrast, administration with HO-1 antagonist SnPP increased cutaneous inflammation, suggesting that HO-1 had a key function in attenuating experimental psoriasis.

This conclusion was consistent with other reports, which showed that the induction of HO-1 also ameliorates psoriasiform skin lesions in the propranolol-induced guinea pig psoriasis model^38^. These results seem to differ from clinical observations because immunohistochemistry revealed prolonged cutaneous HO-1 protein expression accompanied by enhanced Stat3 activation in human psoriatic lesions. This difference may be ascribed to several reasons. First, prolonged cutaneous HO-1 expression likely served as a self-protective response to psoriasis, because HO-1 is a major inducible acute phase protein that can be upregulated by various inducers, such as endotoxin, hydrogen peroxide, prostaglandins, and cytokines. Increased HO-1 expression may be the result of oxidative stress and pro-inflammatory cytokines released during the development of psoriasis. We observed that IL-6/IL-22 treatment *in vitro* failed to trigger the HO-1expression in cultured keratinocyte. This result indicated that prolonged HO-1 overexpression in human psoriatic lesions might be the result of other inflammatory stimuli, such as oxidative stress or TLR ligands. Excess Stat3 activation under prolonged HO-1 overexpression can be the result of the failure of HO-1-mediated protection. Second, the altered HO-1 expression might vary during different phases of disease development, because no information is available regarding the HO-1 expression patterns before and after treatment, or in different severity levels of the disease. Thus, HO-1 expression should be monitored during the entire phase of psoriasis.

This study was the first to show the inhibition of Stat3 signaling by HO-1, and the effects of pharmacological HO-1 induction on the experimental psoriatic mouse model. The *in vivo* protective effect of HO-1 induction on psoriasis was likely ascribed to the inhibition of Stat3 activation in keratinocytes, but other pathological mechanisms may exist. HO-1 has been found to inhibit dendritic cell maturation but conserve IL-10 expression, which may induce a tolerance phenotype as immature dendritic cells; IL-10 can induce the tolerance phenotype by Treg cell induction^39^. The influence of HO-1 on Stat3 inactivation may also interfere with IL-6 expression. IL-6 secretion results in a positive feedback loop, causing further IL-6 production. Stat3 activation is confirmed to be a central event during this process. Thus, the interference of Stat3 signaling by HO-1 induction may easily decrease the IL-6 expression levels.

Our results suggest a protective function of HO-1 induction in psoriasis. The protective effect of HO-1 may be attributed to its effect on the Stat3 signaling pathway via increased SHP-1 expression. This effect prevented Th17 cytokine-induced keratinocyte overproliferation and abnormal keratinocyte differentiation, which indicates that HO-1 induction may be a promising approach in the treatment of cutaneous inflammatory diseases, such as psoriasis.

## Methods

The experimental procedures performed on mice were conducted in accordance with the approved guidelines in the ethical permit approved by the Nanjing University Animal Welfare and Ethics committee. Biopsies and skin from healthy individuals through plastic surgery in this study were obtained from The First Affiliated Hospital of Nanjing University of TCM. Manuscript statements of informed consent were obtained from all donors. This study was approved by the Ethics Committee and Scientific Investigation Board of The First Affiliated Hospital of Nanjing University of TCM, Nanjing, Jiangsu, China.

### Cell lines and tissue samples

Cultured immortalized human keratinocyte (HaCaT) cells (NE Fusenig, Heidelberg, Germany) were obtained from KeyGen Biotech Company. A human epithelial carcinoma cell line (A431), obtained from the American Type Culture Collection (ATCC), was also used in some experiments. Cells were grown in DMEM (Life Technologies, Carlsbad, CA) supplemented with 10% fetal bovine serum (FBS), 100 units/mL penicillin, and 100 μg/mL streptomycin, cultured at 37 °C in 5% CO_2_. When 80% confluence was achieved, HaCaT cells were stimulated using various doses of chemical compounds or cytokines, including 25 ng/mL IL-6, 25 ng/mL IL-22, 25 μM Hemin, and 25 μM CoPP. Human skin biopsies were obtained from patients with psoriasis, and control samples were prepared using skin from healthy individuals through plastic surgery.

### Isolation and culture of primary human keratinocytes

Primary human keratinocytes were obtained from the skin of healthy individuals by plastic surgery. The skin was washed by agitating with forceps in the medium contained Antibiotic-Antimycotic and dispase II in the dish. The tissue was flattened using sterile forceps and was place onto the overturned lid of the culture dish, fat and loose fascia was trimmed away using scissors and forceps. The tissue was rinsed every few minutes to keep the tissue from drying. After the trimming is complete, the tissue was cut into strips approximately 0.5 cm × 1.5 cm using a sterile scalpel and transferred to the tube containing 5 mL of Dispase Solution. After transferred the tube to a 4 °C refrigerator and incubated for 16–21 h at 4 °C. The digested tissue and accompanying Dispase Solution were transferred into the bottom of the culture dish, avoiding splashing. The epidermis was then separated from the dermis. When all the pieces were separated, the epidermal pieces were placed into the tube with 5 mL of EpiLife™ medium containing collagenase and placed at a 37 °C incubator for incubating for 2–4 h. The isolated NHKC were successfully collected and cultured in an EpiLife™ medium containing Antibiotic-Antimycotic.

### Animal models

Eight- to eleven-week-old female BALB/c mice were purchased from the Model Animal Research Center of Nanjing University and raised in an SPF environment. The mice received daily doses of 62.5 mg of IMQ cream (5%) on their shaved backs for 6 days consecutively to induce psoriasis. The control group was similarly treated using a control of Vaseline cream. To investigate the effect of HO-1, mice were randomly divided into five groups (five mice/group): Hemin (5 mg/kg body weight, i.p.) +IMQ group, SnPP (5 mg/kg body weight, i.p.) +IMQ group, DMSO (1% DMSO, i.p.) +IMQ group as a mock control, IMQ group, and control group treated with Vaseline cream. Hemin and SnPP were prepared in DMSO and suspended in PBS, and administered five times every other day starting at one day before IMQ application. DMSO suspended in PBS was injected in the same manner. Mice were euthanized by cervical dislocation after 7 d of IMQ treatment, and skin samples were obtained. Skin samples from the back skin of mice were partially immersed in 4% paraformaldehyde, embedded in paraffin, and cut to 5 μm-thick sections for H&E staining and immunohistochemistry analysis. The other parts of the samples were stored at −80 °C for mRNA extraction.

### Cell proliferation assays

Cytokine-induced keratinocyte proliferation was estimated by the non-radioactive method using a CellTiter 96 Aqueous One Solution Cell Proliferation Assay Kit (Promega®, Madison, WI). A total of 5,000 cells were seeded onto 96-well plates. The culture medium was changed to a medium without FBS after 24 h to equilibrate the cell cycle in the G0/G1phase. After 12–24 h, the cells were incubated with 25 ng/mL IL-6 and IL-22, or 25 μM Hemin for 4 h. After these treatments, we added 100 μL of medium plus 10 μL of MTS-PMS to each well, and the wells were incubated for 90 min. Cell proliferation was determined by measuring the absorbance at 490 nm using an automated ELISA plate reader.

### Western blot

Cells from the different treatment group were collected and washed with PBS, total cell proteins were extracted by incubation for 15 min on ice in RIPA buffer (1% Nonidet P-40, 0.5% sodium deoxycholate, 0.1% SDS in PBS) containing a mixture of protease and phosphatase inhibitors (Thermo Fisher, Waltham, MA). Skin samples were ground before lysis. The proteins were subjected to SDS-PAGE and transferred to PVDF membranes, which were then blocked for 1 h and probed with various primary Abs diluted in PBS containing 5% non-fat dried milk or 3% bovine serum albumin. The membranes were incubated with specific Abs against different proteins overnight at 4 °C. Bound Abs were revealed by chemiluminescence reaction using an ECL-plus detection system (Amersham Pharmacia Biotech), followed by autoradiography.

### Immunohistochemistry

Immunohistochemical staining was performed using standard protocol, and the signals were captured using a PowerVision UltraVision Quanto Detection System (Thermo Scientific) following the manufacturer’s protocol.

### Plasmid and cell transient transfection

Human full-length HO-1 cDNA was amplified by polymerase chain reaction, and subcloned into the GV230 vector to construct recombinant HO-1 expression plasmids GC230-HO-1. HaCaT cells were cultured to near confluence in six-well plates, and transfected with 0.25 mg of GV230-HO-1 plasmid or GV230 control plasmid per well or left untransfected for 6 h for transfection expression. All transfections were performed using Lipofectamine 2000 (Qiagen, Crawley, UK) according to the manufacturer’s recommended protocol. The transfection efficiency was assessed by HO-1 expression analysis using Western blot, according to standard procedures.

### siRNA silencing of gene expression

Chemically synthesized double-stranded siRNA duplexes for HO-1 (5′-ATG GTC TGT AGG GCT TTAT-3′, 5′-GGC CAG CAA CAA AGT GCA A-3′) and SHP-1 (5′-GGG AGG AGA AAG UGA AGA ATT-3′, 5′-UUC UUC ACU UUC UCC UCC CTT-3′) were purchased from Invitrogen (Life Science). HaCaT cells were cultured to 80% confluency in six-well plates, and transfected with 25 μM Hemin and 100 nM specific HO-1 siRNA or control siRNA for 24 h prior to treatment. Time courses and siRNA dose responses were previously studied to determine the optimum conditions for knockdown of target genes. All siRNAs achieved at least a 75% knockdown of target mRNA, and each siRNA was assessed for the induction of Stat3 activation.

### Statistical analyses

Statistical analyses were performed using GraphPad Prism 5.0 (GraphPad Software, San Diego, CA). One-way ANOVA or Student *t*-test was used to test for significant difference. P-values < 0.05 represent significant differences.

## Additional Information

**How to cite this article**: Zhang, B. *et al*. Heme oxygenase-1 induction attenuates imiquimod-induced psoriasiform inflammation by negative regulation of Stat3 signaling. *Sci. Rep.* **6**, 21132; doi: 10.1038/srep21132 (2016).

## Supplementary Material

Supplementary Information
