# Supplementary material for: Heme oxygenase-1 induction attenuates imiquimod-induced psoriasiform inflammation by negative regulation of Stat3 signaling
Source: Sci Rep. 2016 Feb 19;6:21132. doi: 10.1038/srep21132 (PMC4759695; doi:10.1038/srep21132)
Supplement: Supplementary Information [file srep21132-s1.pdf]

Supplementary information

Heme oxygenase-1 induction attenuates imiquimod-induced psoriasiform inflammation by negative regulation of Stat3 signaling

Bin Zhang[1,4], Sijing Xie[1,5], Zhonglan Su[2], Shiyu Song[1], Hui Xu[1], Gang Chen[3], Wangsen Cao[1], Shasha Yin[1], Qian Gao[1]* and Hongwei Wang[1]*

[1]Center for Translational Medicine and Jiangsu Key Laboratory of Molecular Medicine, Medical School of Nanjing University, Nanjing, Jiangsu, 210093, China.

[2]Department of Dermatology, the First Affiliated Hospital of Nanjing Medical University, Nanjing, Jiangsu, 210029, China.

[3]Department of Esthetic Plastic Surgery, The First Affiliated Hospital of Nanjing University of TCM, Nanjing, Jiangsu, 210029, China.

[4]Central Laboratory, Nanjing Chest Hospital, Medical School of Southeast University, Nanjing, Jiangsu, 210029, China.

[5]Nanjing Stomatology Hospital, Medical School of Nanjing University, Nanjing, Jiangsu, 210093, China.

*** Corresponding author: Hongwei Wang and Qian Gao**

Center for Translational Medicine and Jiangsu Key Laboratory of Molecular Medicine, Medical School of Nanjing University, Nanjing, Jiangsu, 210093, China. E-mail: hwang@nju.edu.cn; qian_gao@nju.edu.cn

Tel: +86 25 83594755; Fax: ++86 25 83594755

Supplementary information includes five figures.

Supplementary Figure Legends

Supplementary Figure 1. HO-1 activation in HaCaT cells inhibits Th17 cytokine-induced Stat3 phosphorylation and downstream gene expression. Full scan images of immunoblots for main figures.

Supplementary Figure 2. Altered HO-1 expression levels by recombinant expression or RNAi also influence Stat3 activation in keratinocytes. Full scan images of immunoblots for main figures.

Supplementary Figure 3. HO-1-induced Stat3 inactivation is mediated by SHP-1. Full scan images of immunoblots for main figures.

Supplementary Figure 4. HO-1 inhibited Stat3 activation in normal human keratinocyte through SHP-1 expression. Full scan images of immunoblots for main figures.

Supplementary Figure 5. Therapeutic effect of HO-1 activators on the IMQ-induced psoriasis mouse model. Full scan images of immunoblots for main figures.

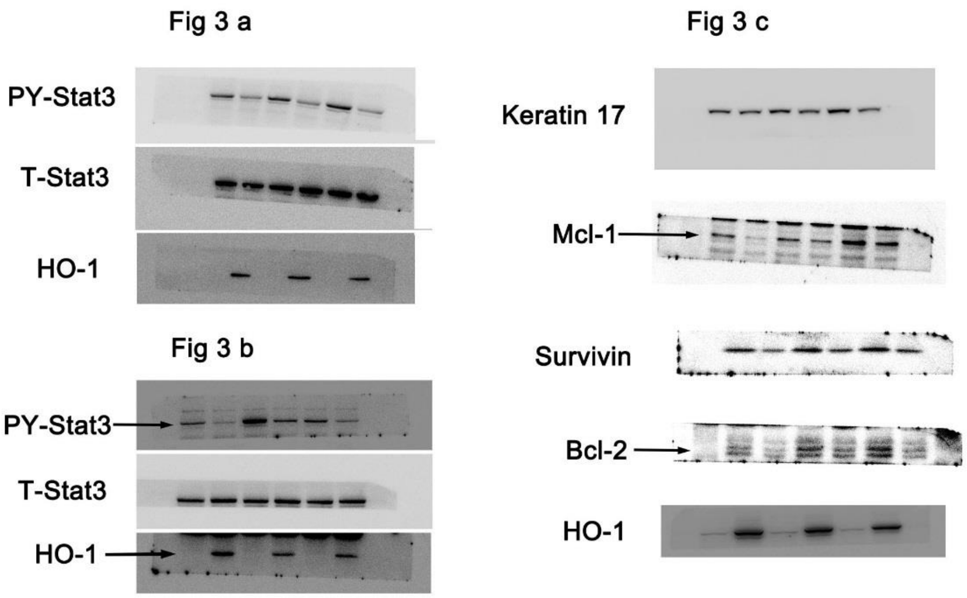

Supplementary Figure 1

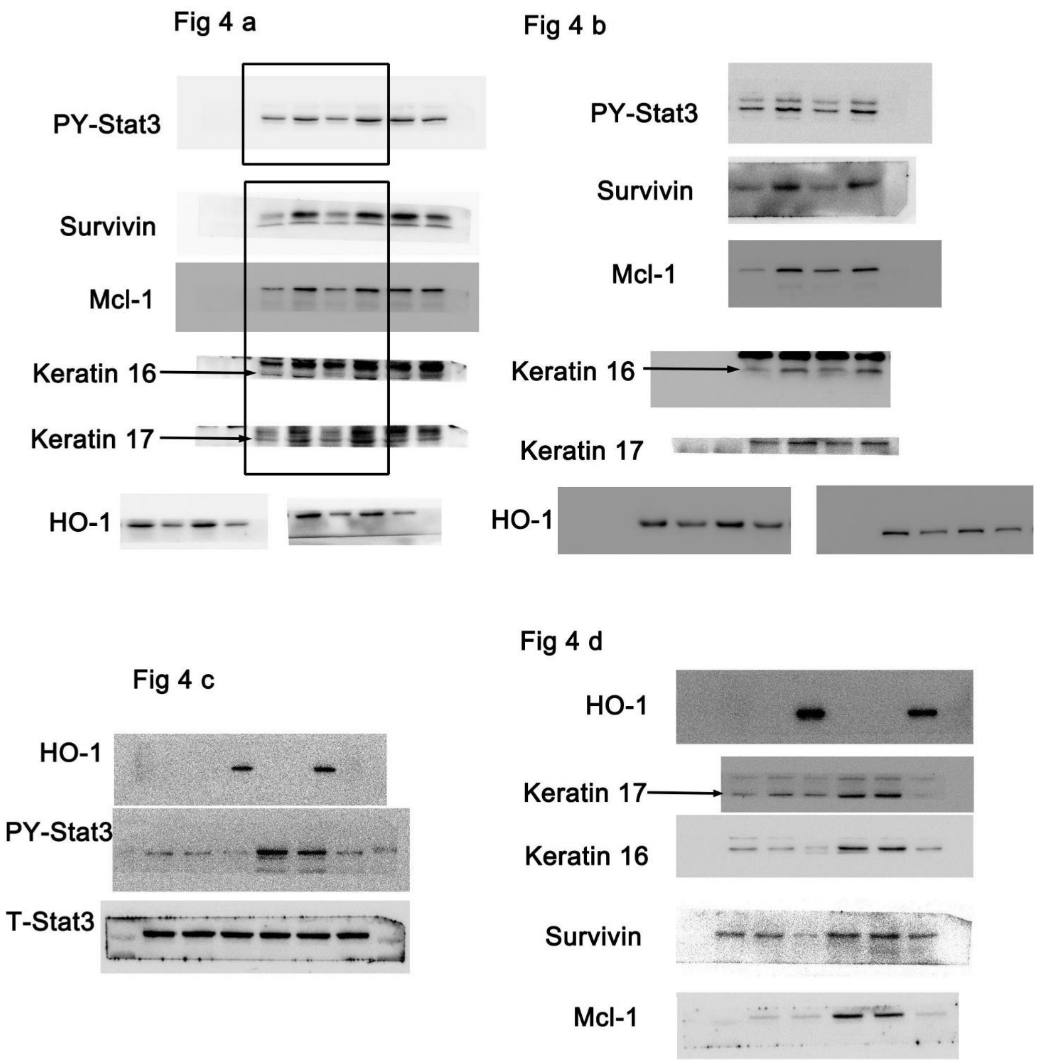

Supplementary Figure 2

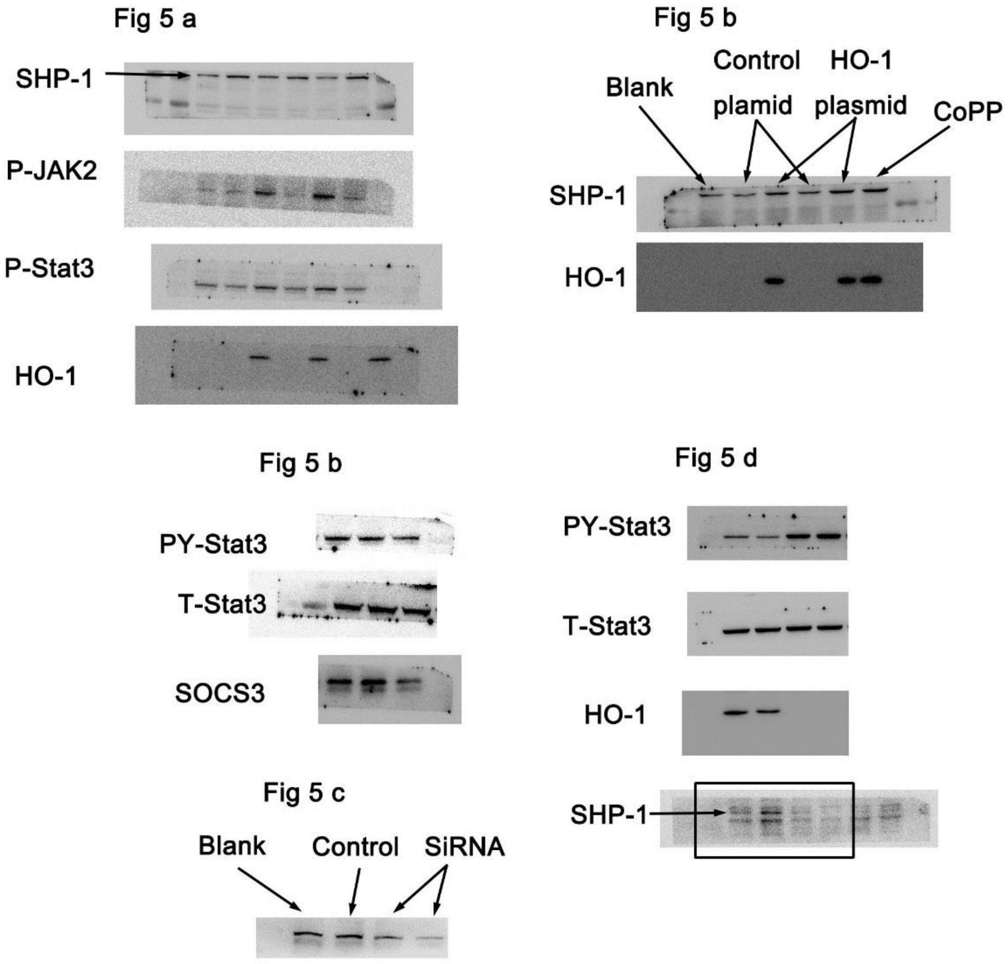

Supplementary Figure 3

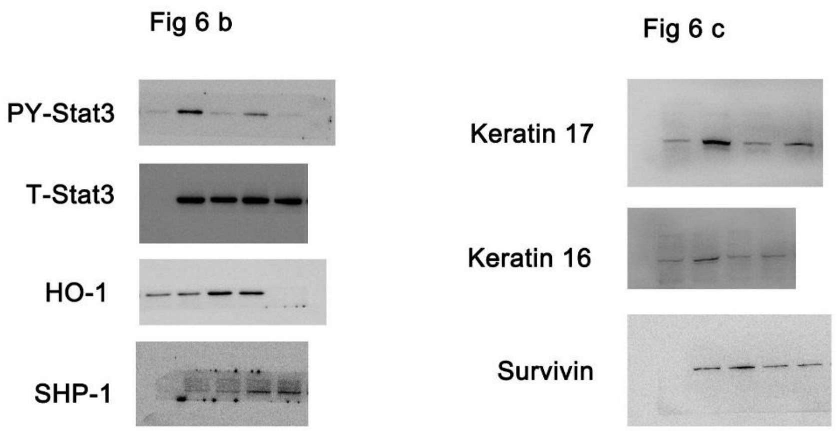

Supplementary Figure 4

Fig 7 c

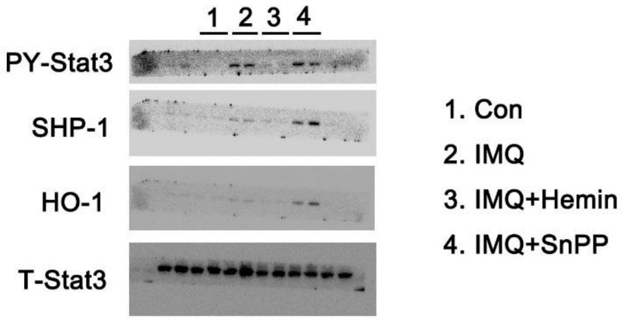

Supplementary Figure 5
